# Supplementary material for: Characterization of Current Husbandry and Veterinary Care Practices of the Giant Pacific Octopus (Enteroctopus dofleini) Using an Online Survey
Source: Vet Sci. 2023 Jul 8;10(7):448. doi: 10.3390/vetsci10070448 (PMC10385140; doi:10.3390/vetsci10070448)
Supplement: Supplementary file 1 [file vetsci-10-00448-s001.zip › vetsci-2457823-supplementary.pdf]

GPO Husbandry and Veterinary Care Survey

Phase 1

1. Are you 18 years old or older?
    a. Yes
    b. No
    If no is selected, will skip to the end of the survey

2. To help us avoid duplication of information from institutions, we ask that you include the name of your institution. As a reminder, no institution names or identifying information will be shared publicly.
    Blank single line

3. Does your institution currently house giant Pacific octopuses (GPOs) (*Enteroctopus dofleini*)?
    a. Yes *Survey skips to question 6*
    b. No *Survey continues to question 4*

4. Is your institution planning to house GPOs in the future?
    a. Yes
    b. Unsure
    c. No

5. Has your institution housed GPOs in the last 5 years?
    a. We HAVE housed GPOs in the last 5 years. *Continues to question 6*
    b. We HAVE housed GPOs, but not within the last 5 years.
    c. We have NEVER housed GPOs.
    d. Unsure
    Answers B, C or D directed to end of survey

6. How many GPOs reside at your institution at the time of this survey?

7. Which best describes your facility/institution's GPO life support system?
    a. Open
    b. Closed
    c. Other/unknown (please comment): ____________

8. What is the average volume of the GPO's habitat (gallons)? Feel free to list dimensions if readily available.

9. For the GPO's water system, please list/describe the average temperature(s) and temperature range(s):

10. Has a GPO at your institution been sedated or anesthetized for any reason (including euthanasia)?

a. Yes
b. Unsure
c. No

11. Would your institution be interested in participating in a more in-depth survey involving questions about GPO husbandry and veterinary care?
    a. Yes
    b. Maybe
    c. No **end of survey**

12. Please provide your name and current email address (or other preferred contact method). Feel free to list questions, comments, or concerns in the space below as well.

End of survey Thank you for your participation. If you expressed interest in completing a followup survey, please be on the lookout for a followup email survey.

Phase 2: **Sent only to willing institutions**

1. Are you 18 years old or older?
    a. Yes
    b. No
       If no is selected, will skip to the end of the survey

2. What is the source for GPOs at your facility? Check all that apply.
    a. Wild caught/collected directly by facility
    b. Purchased commercially immediately from collector/fishery or donated by collector (including bycatch)
    c. Facility transfer
    d. Other (please list):

3. If you acquire GPOs as donations from accidental catch or other unintentional acquisition, how often does this occur? Feel free to include any additional details about types of acquisition.

4. On average, how often is a GPO at your institution fed?

5. Describe the amount and types of food offered:

6. If available, please provide the following information about the life support system (LSS) and habitat:
    a. LSS turnover rate (per hour):
    b. LSS filtration methods:
    c. Tank and decor material:

7. If available, please provide the average (or average range) of additional water chemistry parameters:
    a. pH:
    b. Salinity (ppt):
    c. Nitrate levels (mg/l):
    d. Dissolved oxygen (mg/L):
    e. Water change size (%):
    f. Water change frequency:
    g. Other: ______

8. Do GPOs go through a quarantine period upon entrance?
    a. Yes (note how long): _____
    b. No

9. GPOs at the institution are visually examined by veterinarians at the following times: Check all that apply.
    a. At entrance/new arrival exam
    b. Prior to transfer
    c. Preventative care visual exams
    d. Only when needed
    e. During necropsy
    f. Other ________

10. How often do veterinary visual (or physical) exams occur
    a. Twice a month or more
    b. Every 1 to 2 months
    c. Every 3 to 6 months
    d. Every 7 months to annually
    e. Never to less than once a year

11. Have any procedures EXCLUDING euthanasia been performed on GPOs at your facility in the last two years? Examples of diagnostics include, but are not limited to sample collection (biopsy, hemolymph collection), imaging, surgery, device implantation, wound repair, etc.)
    a. Yes *Continues to question 12 and 13*
    b. No *Skips to question 14*
    c. Unsure *Skips to question 14*

12. Briefly describe or list procedures:

13. Was sedation or anesthesia used?
    a. Yes
    b. Unsure
    c. No

14. Does your institution use cooling or freezing for immobilization or euthanasia of GPOs?
    a. Yes, as a second step following chemical sedation/anesthesia
    b. Yes, as the sole means of immobilization
    c. Unsure
    d. No

    Either Yes proceeds to 15, No/unsure skips to 16

15. Describe procedure including temperatures reached and general time frames:

16. Does your institution use the following for sedation or anesthesia of GPOs: Select all that apply.
    a. Magnesium chloride
    b. Magnesium sulfate
    c. Ethanol
    d. Isoflurane
    e. Sevoflurane
    f. Benzocaine
    g. MS-222
    h. Eugenol
    i. Other - please list ___

17. Please describe, or attach, sedation or anesthesia procedures at your institution including dosages, general expected time frame, and/or any other helpful observations.

18. Has your facility euthanized GPOs?
    a. Yes
    b. Unsure

c. No

19. Does your institution use the following for euthanasia of GPOs: Select all that apply
    a. Magnesium chloride
    b. Magnesium sulfate
    c. Ethanol
    d. Isoflurane
    e. Sevoflurane
    f. Benzocaine
    g. MS-222
    h. Eugenol
    i. Potassium chloride
    j. Pentobarbital
    k. Mechanical decerebration
    l. Other - list

20. Please describe, or attach, euthanasia protocols at your institution including dosages, general expected time frame, and/or any other helpful observations:

21. How do you determine that the appropriate level of sedation/anesthesia/euthanasia has been achieved?

22. Have you or your institution had any negative experiences using any of the following with GPOs: Check all that apply. Please describe these negative experiences.
    a. Magnesium chloride ________
    b. Magnesium sulfate ________
    c. Ethanol ________
    d. Isoflurane ________
    e. Sevoflurane ________
    f. Benzocaine ________
    g. MS-222 ________
    h. Eugenol ________
    i. Potassium chloride ________
    j. Pentobarbital ________
    k. Decerebration _______
    l. Cooling ________
    m. Other. Please list and describe. ________

23. Would you potentially be willing to allow us to view your facility's medical records?
    a. No
    b. Maybe
    c. Yes (please describe the process needed) ______

24. Thank you for your participation! Feel free to leave questions or additional comments here.

End of survey
